# The fluorochrome-to-protein ratio is crucial for the flow cytometric detection of tissue factor on extracellular vesicles

**DOI:** 10.1038/s41598-024-56841-5

**Published:** 2024-03-17

**Authors:** René Weiss, Marwa Mostageer, Tanja Eichhorn, Silke Huber, Dominik Egger, Andreas Spittler, Carla Tripisciano, Cornelia Kasper, Viktoria Weber

**Affiliations:** 1 Center for Biomedical Technology, Department for Biomedical Research, University for Continuing Education Krems, Dr.-Karl-Dorrek-Strasse 30, 3500 Krems, Austria; 2 Institute of Hygiene and Medical Microbiology, Medical University of Innsbruck, Innsbruck, Austria; 3 Institute of Cell and Tissue Culture Technology, Department of Biotechnology, University of Natural Resources and Life Sciences, Vienna, Austria; 4 Core Facility Flow Cytometry & Surgical Research Laboratories, Medical University of Vienna, Vienna, Austria; 5 Clinical Division of Haematology and Haemostaseology, Department of Medicine I, Medical University of Vienna, Vienna, Austria

**Keywords:** Extracellular vesicles, Flow cytometry, Fluorochrome-to-protein ratio, Tissue factor, Coagulation system, Mesenchymal stem cells, Monocytes and macrophages

## Abstract

Extracellular vesicles (EVs) have crucial roles in hemostasis and coagulation. They sustain coagulation by exposing phosphatidylserine and initiate clotting by surface expression of tissue factor (TF) under inflammatory conditions. As their relevance as biomarkers of coagulopathy is increasingly recognized, there is a need for the sensitive and reliable detection of TF^+^ EVs, but their flow cytometric analysis is challenging and has yielded controversial findings for TF expression on EVs in the vascular system. We investigated the effect of different fluorochrome-to-protein (F/P) ratios of anti-TF-fluorochrome conjugates on the flow cytometric detection of TF^+^ EVs from activated monocytes, mesenchymal stem cells (MSCs), and in COVID-19 plasma. Using a FITC-labeled anti-TF antibody (clone VD8), we show that the percentage of TF^+^ EVs declined with decreasing F/P ratios. TF was detected on 7.6%, 5.4%, and 1.1% of all EVs derived from activated monocytes at F/P ratios of 7.7:1, 6.6:1, and 5.2:1. A similar decline was observed for EVs from MSCs and for EVs in plasma, whereas the detection of TF on cells remained unaffected by different F/P ratios. We provide clear evidence that next to the antibody clone, the F/P ratio affects the flow cytometric detection of TF^+^ EVs and should be carefully controlled.

## Introduction

There is ample evidence for the role of circulating extracellular vesicles (EVs) in coagulation and for their involvement in various pathologies associated with thromboembolic events, such as atherosclerosis, cancer, sepsis, or severe COVID-19^1–4^. Plasma membrane-derived EVs support coagulation via the exposure of negatively charged phospholipids, such as phosphatidylserine (PS), which provide a catalytic surface for the formation of the tenase (coagulation factors VIIIa, IXa, and X) and prothrombinase (factors Va, Xa, and II) complexes of the coagulation cascade^5,6^, and enhance their activities by up to three orders of magnitude^5,7,8^. While the exposure of PS supports the propagation of coagulation, the initiation of the coagulation cascade depends on active tissue factor (TF; CD142), which is the receptor for coagulation factors VII/VIIa and the primary initiator of coagulation in vivo. TF is not expressed in the vascular system under physiological conditions but is induced on monocytes and neutrophils as well as on endothelial cells under inflammatory conditions. Neutrophils externalize TF on neutrophil extracellular traps (NETs) during atherothrombosis^9,10^, and the generation of NETs decorated with TF is a driver of immunothrombosis in sepsis and severe COVID-19^11^. Likewise, TF is expressed by tumor cells, where it contributes to metastasis and angiogenesis^12^. TF can be released from the cell surface and spread in the vascular system in the form of TF positive (TF^+^) EVs. Accordingly, EVs are essential for both, initiating and propagating coagulation in the vascular system.

While TF expression on activated monocytes, neutrophils, and endothelial cells is well established in inflammatory pathologies^13–15^, the presence of TF on platelets and on platelet-derived circulating EVs remains controversial^16–20^. It has been suggested that the use of different methodological approaches and of different antibody clones for TF detection on cells or EVs may at least in part account for these divergent results^18,21^.

Flow cytometry is a versatile technique that is widely used for the phenotypic characterization of cells and EVs^22–24^. The immunodetection of proteins on EVs using fluorochrome-conjugated antibodies poses several methodological challenges that are related to the small size of EVs^22,23,25^. The number of epitopes on the EV surface and thus the number of fluorochrome-antibody conjugates which can bind to this surface decreases quadratically with decreasing diameter. Consequently, the lower number of epitopes on EVs in relation to their cells of origin results in lower signal intensity. Furthermore, depending on the size of the conjugated fluorochromes, steric hindrance may limit the binding of fluorochrome-antibody conjugates to the EV surface^21^, and the membrane curvature can affect the conformation of epitopes and have an impact on the binding efficiency of fluorochrome-antibody conjugates to their target molecules^26^. Differences in the affinity of antibody clones for their target epitopes have been reported to influence flow cytometric EV characterization, as well^21^. Finally, batch-to-batch variations during the production of fluorochrome-antibody conjugates may result in different fluorochrome-to-protein (F/P) ratios and thereby influence the signal intensity^22,23,25,27–29^.

Here, we investigated the effect of different F/P ratios of anti-TF fluorochrome-antibody conjugates on the detection of TF on PS-positive EVs from different sources (activated monocytes, mesenchymal stem cells, COVID-19 plasma samples). We show for the first time that the F/P ratio of fluorochrome-antibody conjugates is critical for the detection of TF on EVs, while it does not affect the detection of TF on cells.

## Results

### The detection of TF on lipopolysaccharide (LPS)-stimulated monocytes and mesenchymal stem cells (MSCs) is unaffected by the antibody clone and by the F/P ratio

**Figure 1:**
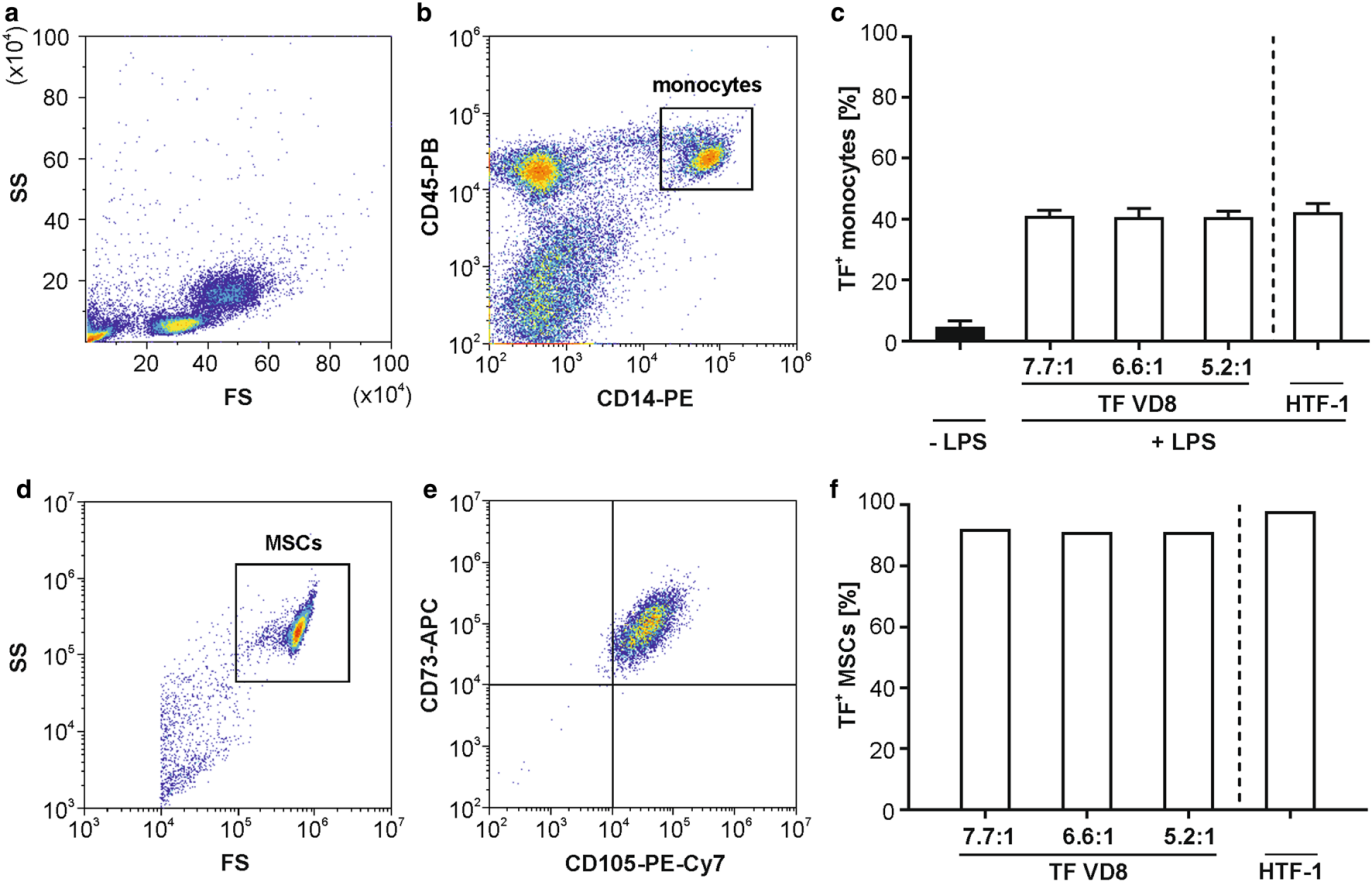
Flow cytometric characterization of TF expression on monocytes and MSCs. (**a**) Monocytes were enriched from PBMCs as described in the “Methods” section, and (**b**) stained with PB-conjugated anti-CD45 as leukocyte marker and with PE-conjugated anti-CD14 as monocyte marker. Monocytes were identified as CD45^+^CD14^+^ cells. (**c**) Monocytes were stimulated with 10 ng/mL LPS for 6 h (+ LPS) or left untreated (− LPS), and TF expression was assessed by staining with FITC-conjugated anti-TF (clone VD8) at different fluorochrome-to-protein (F/P) ratios as indicated, or with PE-conjugated anti-TF (clone HTF-1). TF expression of untreated monocytes was assessed by staining the cells with FITC-conjugated anti-TF (clone VD8) F/P ratio 7.7:1. (**d**) MSCs were isolated as described in the “Methods” section, and (**e**) stained with APC-conjugated anti-CD73 and PE-Cy7-conjugated anti-CD105. (**f**) TF was detected with FITC-conjugated anti-TF (clone VD8) at different F/P ratios as indicated, or with PE-conjugated anti-TF (clone HTF-1). n = 3 for monocytes; n = 1 for MSCs.

LPS-stimulated monocytes and MSCs were probed for TF expression after staining with the FITC-conjugated anti-TF antibody clone VD8 (F/P ratios 5.2:1; 6.6:1; 7.7:1) and the PE-conjugated anti-TF antibody clone HTF-1 (F/P ratio not specified by the supplier) as shown in Fig. 1. Both, LPS-stimulated monocytes and MSCs expressed TF (41.2 ± 0.8% *vs*. 93.0 ± 3.4% positive cells). TF expression on monocytes reached its maximum between 6 and 8 h post LPS stimulation (Supplementary Fig.S1 online). The percentage of TF^+^ cells did neither differ between the two antibody clones, nor did different F/P ratios affect the detection of TF on cells (Fig. 1c,f).

### The TF signal on EVs derived from LPS-stimulated monocytes or from MSCs critically depends on the F/P ratio

**Figure 2:**
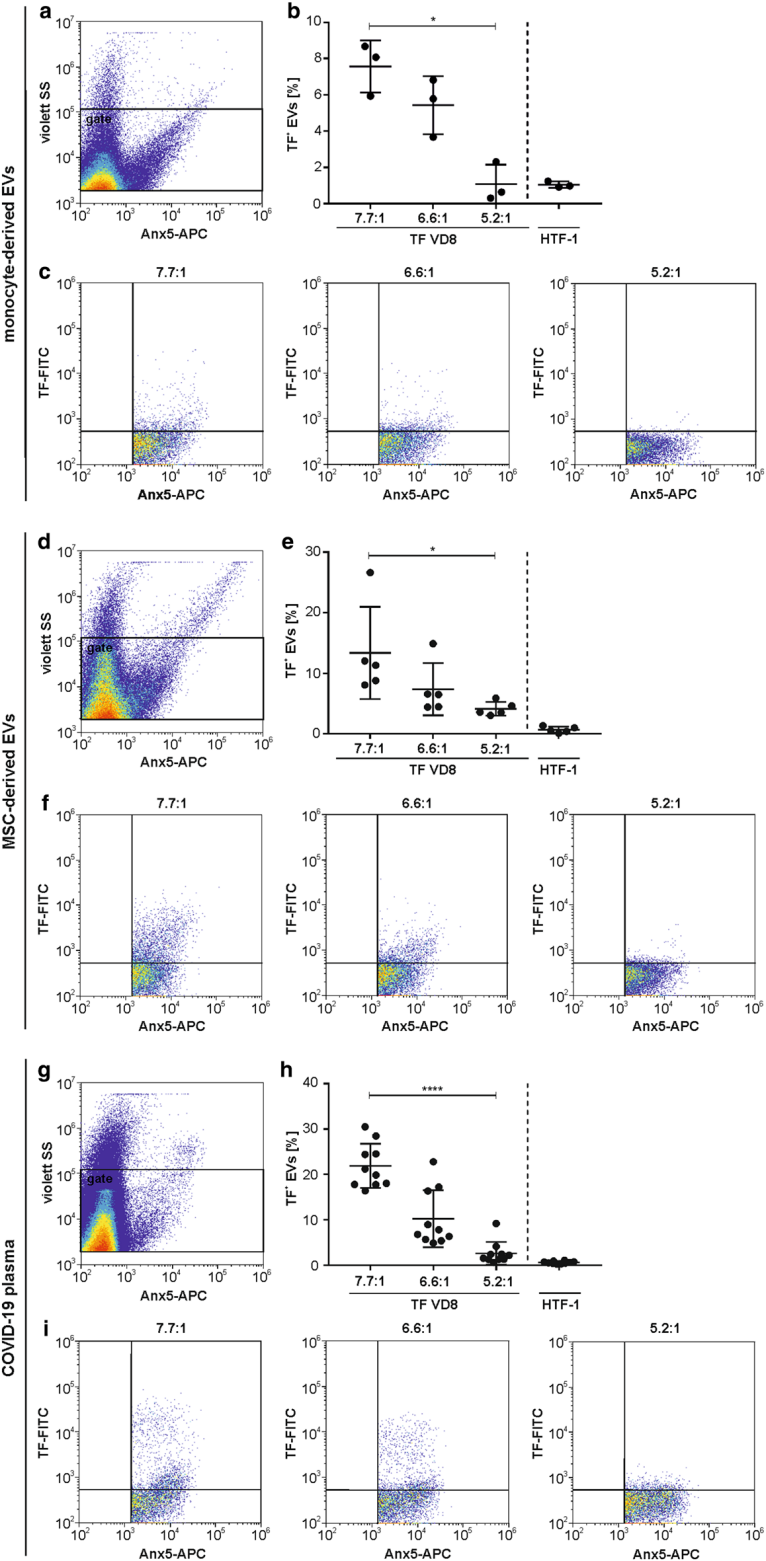
Flow cytometric characterization of TF-expressing phosphatidylserine-exposing EVs derived from monocytes, MSCs, and COVID-19 plasma. EVs were stained with APC-conjugated Anx5 as marker for phosphatidylserine and with FITC-conjugated anti-TF antibody (clone VD8) or with PE-conjugated anti-TF antibody (clone HTF-1). A representative plot of Anx5 *vs*. violet SS is shown for (**a**) monocyte-derived EVs, (**d**) MSC-derived EVs, and (**g**) EVs from COVID-19 plasma. Percentages of TF-expressing EVs from (**b**) monocytes (n = 3), (**e**) MSCs (n = 5), and (**h**) plasma (n = 10) obtained at different fluorochrome-to-protein (F/P) ratios are depicted (*p < 0.05; ****p ≤ 0.0001). Percentages [%] refer to all Anx5^+^ events. Representative TF (clone VD8; F/P ratios as indicated) *vs*. Anx5 plots are shown for (**c**) monocyte-derived EVs, (**f**) MSC-derived EVs, and **(i)** EVs from COVID-19 plasma.

Next, we assessed the exposure of TF on PS-positive EVs derived from LPS-stimulated monocytes and from MSCs and found that the detection of TF on EVs was strongly affected by the F/P ratio of FITC-labeled VD8. TF was detected on 7.6 ± 1.4% *vs*. 5.4 ± 1.6% *vs*. 1.1 ± 1.1% of all EVs derived from LPS-stimulated monocytes when using F/P ratios of 7.7:1 *vs.* 6.6:1 *vs.* 5.2:1, respectively (Fig. 2, upper panel). Likewise, the percentage of TF^+^ MSC-derived EVs declined with decreasing F/P ratios (13.4 ± 7.6% *vs*. 7.4 ± 4.3% *vs*. 4.1 ± 1.1% for F/P ratios of 7.7:1 *vs.* 6.6:1 *vs.* 5.2:1, respectively; Fig. 2, middle panel). The anti-TF clone HTF-1 was less efficient in detecting TF on EVs from stimulated monocytes or from MSCs, yielding only 1.0 ± 0.2% and 0.7 ± 0.5% TF^+^ EVs, respectively (Fig. 2b,e). For all F/P ratios, the percentage of TF^+^ EVs correlated with the TF expression of their cells of origin. Hence, the higher percentage of TF^+^ MSCs (93.0 ± 3.4%) as compared to TF^+^ monocytes (41.2 ± 0.8%) was mirrored by a higher percentage of TF^+^ MSC-derived EVs (13.4 ± 7.6%) as compared to EVs from LPS-stimulated monocytes (7.6 ± 1.4%). Concentrations of total EVs and TF^+^ EVs for monocytes and MSCs are given in Supplementary Table S1 online and Supplementary Table S2 online, respectively.

### Detection of TF on circulating EVs from COVID-19 patients

In addition to using monocyte- and MSC-derived PS-positive EVs, we characterized TF expression of EVs in clinical samples from patients suffering from severe COVID-19. Of the 134 plasma samples (see “Methods” section), we selected 10 samples with > 15% of TF^+^ EVs, characterized with the FITC-conjugated VD8 (F/P ratio 7.7:1). We re-analyzed these samples with the same antibody clone, but at different F/P ratios (7.7:1 *vs.* 6.6:1 *vs.* 5.2:1) as well as with the PE-conjugated HTF-1 (Fig. 2, lower panel). Plasma samples contained 8.8 × 10^4^ ± 7.0 × 10^4^ EVs/µL, defined as Anx5-binding events in the EV gate, as described in the “Methods” section. Staining with FITC-conjugated VD8 detected TF expression on 21.9 ± 4.9%, 10.2 ± 6.3%, and 2.6 ± 2.5% of all EVs for F/P ratios of 7.7:1, 6.6:1, and 5.2:1, respectively, and on 0.6 ± 0.3% with PE-conjugated HTF-1 (Fig. 2h). Concentrations of total EVs and TF^+^ EVs for COVID-19 plasma are listed in Supplementary Table S3 online. The majority of EVs in COVID-19 patients originated from platelets (30.9 ± 17.6%, CD41^+^ EVs) followed by red blood cells (10.1 ± 7.1%, CD235a^+^ EVs) and leukocytes (9.4 ± 3.5%, CD45^+^ EVs).

### Competition assays

**Figure 3:**
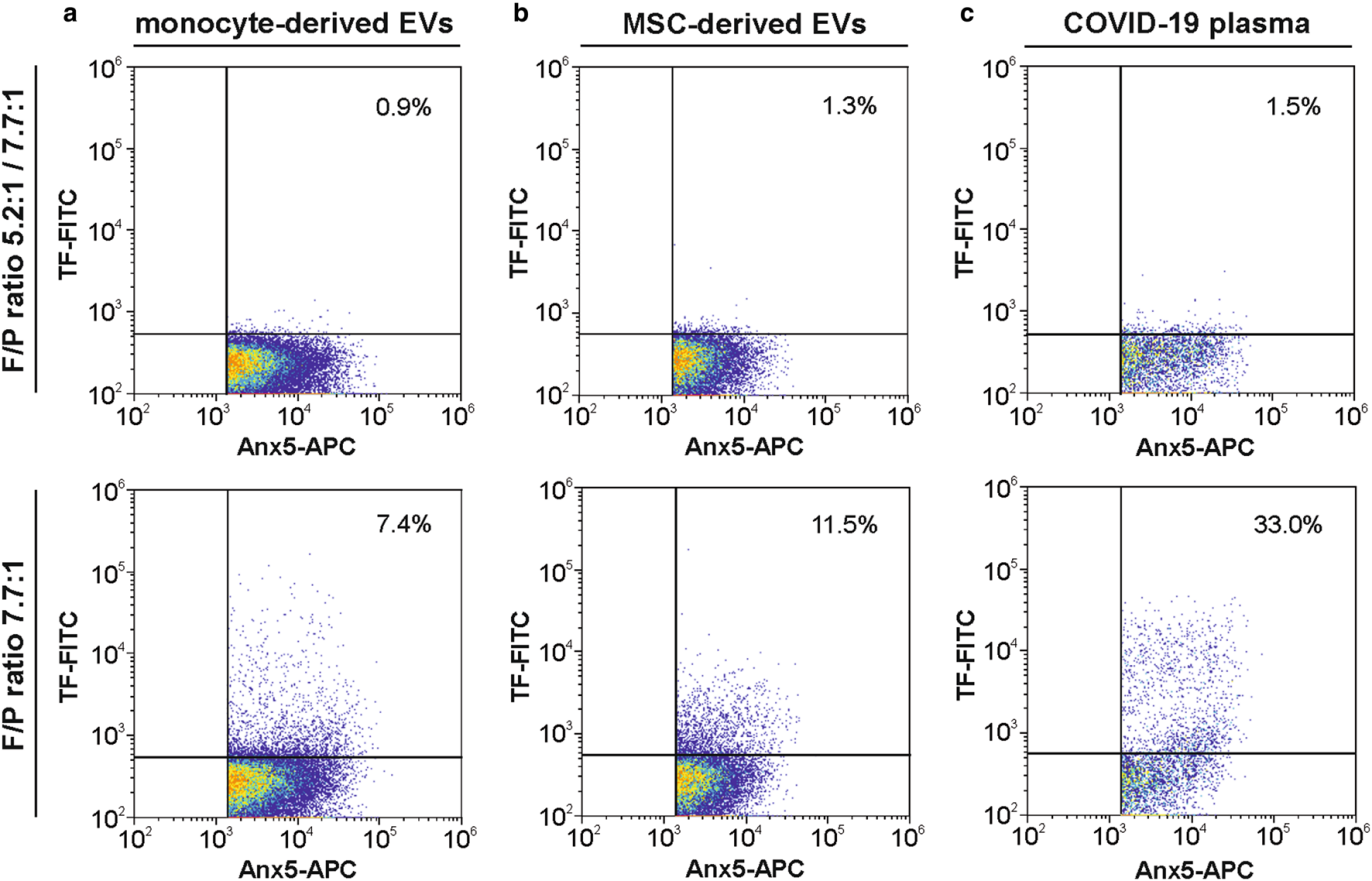
Competition assay. (**a**) Monocyte-derived and (**b**) MSC-derived EVs, as well as (**c**) EVs in plasma from COVID-19 patients were pre-stained with FITC-conjugated anti-TF (clone VD8, F/P ratio 5.2:1) followed by staining with anti-TF (clone VD8, F/P ratio 7.7:1; upper panel). Representative TF *vs.* Anx5 plots are shown. The lower panel represents EVs stained with FITC-conjugated anti-TF (clone VD8, F/P ratio 7.7:1) only.

Pre-staining of EVs derived from activated monocytes, from MSCs, or from plasma samples with FITC-conjugated VD8 (F/P ratio 5.2:1) followed by staining at a higher F/P ratio (7.7:1) yielded less than 1.5% of TF^+^ EVs (Fig. 3). This indicates that almost all TF epitopes were occupied by VD8 (F/P ratio 5.2:1) in the pre-staining step and further excludes unspecific binding of VD8 (F/P ratio 7.7:1).

## Discussion

Flow cytometry is widely applied to characterize EVs in complex samples such as human whole blood, despite considerable methodological challenges related to their small size and heterogeneity.

To assess whether previously reported controversial flow cytometry data on TF expression on EVs could be related to the use of different antibody clones and/or different F/P ratios, we investigated TF expression on PS-positive EVs using two different anti-TF fluorochrome conjugates, FITC-conjugated VD8 and PE-conjugated HTF-1. TF is a 263 amino acid transmembrane protein comprising an extracellular domain of 219 amino acids, a 23 amino acid transmembrane domain, and a 21 amino acid intracellular domain^30^. VD8 recognizes an epitope comprising amino acids 1–25 of TF^31^, whereas the epitope targeted by HTF-1 consists of Tyr94 and Phe76^32^. Using these two clones, we analyzed TF expression on LPS-stimulated monocytes and monocyte-derived EVs, as well as on MSCs and MSC-derived EVs. TF^+^ monocytes and monocyte-derived EVs were selected due to their clinical relevance, as they are centrally involved in the progression of immunothrombosis in sepsis and severe COVID-19^33,34^. MSCs and MSC-derived EVs were chosen since the reliable characterization of their TF expression is crucial to avoid or limit side-effects during clinical application^35^. As a third example, we characterized EVs in clinical plasma samples from patients suffering from severe COVID-19.

We found that only clone VD8 was able to recognize TF on EVs, whereas no positive signal was obtained with clone HTF-1. This confirms previous findings that the choice of different antibody clones plays a significant role in detecting TF on EVs^21^. When comparing different commercial anti-TF antibody clones in flow cytometry, Basavaraj and co-workers found that only two out of five clones, among them VD8, recognized TF on cells and on EVs, whereas three clones failed to detect TF on EVs.

Differences in the detection of surface molecules on cells *vs*. EVs have been associated with differences in the membrane curvature, which may influence the accessibility and the conformation of certain epitopes. As an example, there are indications that Anx5 preferentially binds to PS on surfaces with low curvature, whereas lactadherin efficiently binds to PS-exposing membranes regardless of their curvature^26^. For the interpretation of our data, we might assume that VD8 is less sensitive to the effect of membrane curvature because it targets a linear epitope of 25 amino acids, whereas HTF-1 is directed against a conformational epitope of only two amino acids.

Diagnostic antibodies are commonly developed for the characterization of cells, which are approximately ten times larger in diameter as compared to EVs. In planar configuration, an EV with a diameter of 100 nm can bind approximately 4000 antibody molecules with a diameter of 3 nm, 100 times less than a cell of 10 µm in diameter, as their surface area is 10,000-fold smaller^36^. The lower number of epitopes on EVs as compared to cells cannot fully be compensated by a larger number of fluorochrome molecules per antibody, due to the phenomenon of quenching, where intermolecular interactions and energy transfer between adjacent molecules result in reduced quantum yield^37^. Furthermore, the conjugation of antibodies with fluorescent labels has been shown to decrease their avidity. Thus, it is crucial to meet the balance between preserving the functionality of an antibody and providing a sufficient degree of fluorescent labeling during manufacturing of the antibody-fluorochrome conjugates^38^.

We hypothesized that next to the antibody clone, the fluorochrome-to-protein ratio might have an impact on the detection of TF on EVs, and indeed, an increasing F/P ratio resulted in the detection of an increased number of Anx5^+^TF^+^ EVs, whereas it had no influence on the detection of TF on cells, confirming that antibodies with an optimized F/P ratio are crucial for the detection of TF on EVs.

Our study has several limitations. Information on the F/P ratio of the PE-labeled anti-TF antibody clone HTF-1 was not provided by the supplier. Therefore, we cannot make any statement as to whether TF would be detectable on EVs when using PE-labeled HTF-1 at a different F/P ratio. However, our findings confirm previous data on the lack of binding of HTF-1 to TF on EVs^21^.

As only FITC-labeled VD8 was available, we are not able to extend our findings to other fluorochromes, and our results have not yet been extrapolated to other EV surface molecules. Moreover, the limitations related to the flow cytometric characterization of EVs should be taken into account. There is evidence that Anx5, which we used to detect PS-exposing EVs in this study, also labels apolipoprotein B-containing lipoproteins, such as low-density lipoprotein^39^, challenging the use of Anx5 to uniquely identify EVs in lipoprotein-containing samples. In fact, the Anx5^+^ events identified with our protocol may include lipoprotein particles, as well. This is particularly true for EVs from plasma samples, whereas the presence of lipoproteins in monocyte- and MSC-derived EVs is less likely. The former were obtained from monocytes isolated from whole blood using a multistep protocol including numerous washing steps, and MSC-derived EVs were isolated from cell culture samples. Still, we cannot fully rule out the presence of lipoproteins in our samples. As we obtained comparable results with all three groups of samples (EVs derived from monocytes, MSCs, and plasma), however, it is unlikely that our results were confounded by lipoproteins.

Another limitation of this study is the presentation of fluorescence intensities in arbitrary units and the lack of indication of the triggering threshold and applied gates in standardized units (e.g., MESF; molecules of equivalent soluble fluorochrome). When the present study was initiated, standardization was hardly an issue, but future studies will be performed under standardized conditions. In particular, the limitations relate to the reproducibility of flow cytometer results due to different optical sensitivity, alignment and electronic noise on the detector, which contributes to different quantum detection efficiencies and the abilities to separate signal from noise. In addition, standardization also enables reproducibility and includes the refractive index distribution of EVs. To address this limitation in future studies, it is important that follow-up studies in the field of EV research calibrate the flow cytometer signals to MESF and size. This crucial calibration step, described in detail by Welsh and co-authors, will both standardize own results and greatly improve reproducibility and comparability when using different flow cytometers^23,40–42^.

Despite all limitations, we can conclude that the detection of TF on EVs critically depends on the F/P ratio of the antibody-fluorochrome conjugate, whereas it does not affect the detection of TF on cells. To our knowledge, the impact of the F/P ratio on surface marker detection has not been systematically analyzed before, while it obviously affects the interpretation of flow cytometry data. Given the biological significance of TF expression particularly in the clinical setting, the F/P ratio should therefore be carefully considered and indicated for different batches by the suppliers of anti-TF fluorochrome conjugates.

## Methods

### Human whole blood

Whole blood was drawn from healthy volunteer donors into Vacuette tubes (Greiner Bio-One, Kremsmuenster, Austria) containing sodium citrate using a 21-gauge needle without tourniquet application (Greiner Bio-One). The first aliquot was used for blood cell counting (Sysmex KX-21 N, Sysmex, Neumünster, Germany). The study was approved by the Ethical Review Board of the University for Continuing Education Krems (EK GZ 13/2015-2018, first date of approval: January 14, 2013; renewed on March 22, 2022), and written informed consent was obtained from all donors.

### Chemicals and reagents

The Pan Monocyte Isolation Kit was purchased from Miltenyi Biotec, Bergisch Gladbach, Germany. Lipopolysaccharide (LPS) from *E. coli* (055:B5), RPMI-1640 medium, 4-(2-hydroxyethyl)-1-piperazineethanesulfonic acid (HEPES), human AB serum, ethylenediaminetetraacetic acid disodium salt (EDTA), penicillin/streptomycin, and accutase were from Sigma Aldrich (St. Louis, MO). Ficoll-Paque PLUS medium was purchased from GE Healthcare (Uppsala, Sweden), MEM (minimal essential medium) α was from Thermo Fisher Scientific (Waltham, MA), human platelet lysate from PL BioScience (Aachen, Germany), gentamycin from Lonza (Basel, Switzerland), and heparin from Ratiopharm (Ulm, Germany). Dulbecco’s phosphate buffered saline (DPBS) with (+/+) or without (−/−) calcium and magnesium was obtained from Life Technologies (Paisley, UK), and annexin V (Anx5) binding buffer was purchased from BD Biosciences (San Jose, CA).

### Isolation and stimulation of primary human monocytes

Freshly drawn human whole blood (40 mL) was diluted 1:2 in DPBS^−/−^ containing 5 mM EDTA, and peripheral blood mononuclear cells (PBMCs) were enriched by density gradient centrifugation on Ficoll-Paque PLUS medium as previously described^43^. Monocytes were isolated from PBMCs using the Pan Monocyte Isolation Kit according to the instructions of the manufacturer (Miltenyi Biotec). This protocol is based on the labeling of non-monocytes with biotin-conjugated antibodies and the subsequent depletion of labeled cells by binding to streptavidin-conjugated magnetic beads, yielding “untouched” monocytes.

Freshly isolated monocytes were resuspended in RPMI-1640 medium, supplemented with 20 mM HEPES, 100 IU/mL penicillin, 100 µg/mL streptomycin, and 10% human AB serum (centrifuged at 20,000 g for 30 min at 4 °C and 0.2 µm sterile filtered using a Minisart syringe filter, Sartorius Stedim Biotech, Goettingen, Germany). Cells were seeded à 1 mL into 24-well plates (Greiner Bio-One) at a density of 1 × 10^6^ cells/mL and stimulated with 10 ng/mL LPS for 2, 4, 6, 8, and 19 h in humidified atmosphere (37 °C, 5% CO_2_; Supplementary Fig. S1 online). For all further experiments, monocytes were stimulated for 6 h. Untreated monocytes served as control. The cell suspension was collected, and monocytes were pelleted at 500×*g* (15 min, room temperature) for flow cytometric characterization (see below). The supernatant was centrifuged (2500×*g*, 15 min, 4 °C) to remove cell debris, aliquoted and frozen at – 80 °C until EV isolation. EVs were pelleted from 500 µL supernatant at 20,000×*g* (30 min, 4 °C), resuspended in 50 µL and analyzed by flow cytometry as described below. A detailed workflow on the isolation of monocyte-derived EVs is shown in Supplementary Fig. S2 online. Relevant EV isolation controls (size distribution and particle concentration) were determined using Nanoparticle Tracking Analysis (NTA; ZetaView, PMX-110, Particle Metrix, Inning, Germany) and are summarized in Supplementary Table S4 online.

### Flow cytometric analysis of TF expression on monocytes

To analyze TF expression on monocytes, 1 × 10^5^ of LPS-stimulated or unstimulated cells in 100 µL DPBS^+/+^ were stained with PE or PB-conjugated anti-CD14 (final staining concentration is 41 ng/mL for panels VD8 or 3.3 µg/mL for HTF-1), PB-conjugated anti-CD45 (final staining concentration 4.1 µg/mL), APC-conjugated anti-CD66b (final staining concentration is 517 ng/mL for the VD8 panel or 488 ng/mL for HTF-1), PE-Cy7-conjugated CD41 (final staining concentration 413 ng/mL for VD8 panel or 391 ng/mL for HTF-1), and FITC-conjugated anti-TF (clone VD8, isotype G1, final staining concentration 4.1 µg/mL) at different fluorochrome-to-protein (F/P) ratios (7.7:1; Lot: 190402 *vs*. 6.6:1; Lot: 180215 *vs*. 5.2:1; Lot: 200708) or with PE-conjugated anti-TF (clone HTF-1; isotype IgE1, final concentration 1.9 µg/mL; Lot: 0030902; BD Biosciences, Franklin Lakes, New Jersey; F/P ratio not provided by the supplier) for 30 min in the dark on ice. All fluorochrome-labeled antibodies are specified in Table 1 and detailed staining protocols are given in Supplementary Table S5 online. Stained monocytes were diluted 1:3 in DPBS^+/+^ and analyzed on a CytoFLEX LX flow cytometer (Beckman Coulter) equipped with 375 nm, 405 nm, 488 nm, 561 nm, and 638 nm lasers using the gating strategy shown in Fig. 1. Data were acquired for 3 min at a flow rate of 30 μL/min and analyzed using the Kaluza Software 2.1 (Beckman Coulter).

**Table 1:** Antibodies and fluorochrome conjugates used for flow cytometry.

Flow cytometry							
Antigen	Origin	Clone	Marker for	Fluorochrome	Abbreviation	Supplier	Cat. #
CD73	Mouse	AD2	MSCs	Allophycocyanin	APC	Invitrogen	17073942
CD105	Mouse	SN6	MSCs	Phycoerythrin–cyanine 7	PE-Cy7	Invitrogen	25105742
CD14	Mouse	RMO52	Monocytes	Phycoerythrin	PE	Beckman Coulter	A07764
CD14	Mouse	63D3	Monocytes	Pacific blue	PB	BioLegend	367121
CD45	Mouse	J33	Leukocytes	Pacific blue	PB	Beckman Coulter	A74763
CD66b	Mouse	80H3	Granulocytes	Allophycocyanin	APC	Beckman Coulter	B15091
CD41	Mouse	P2	Platelets	Phycoerythrin–cyanine 7	PE-Cy7	Beckman Coulter	6607115
CD235a	Mouse	HIR2	Red Blood Cells	Fluorescein isothiocyanate	FITC	Invitrogen	11998782
TF	Mouse	VD8	Tissue Factor	Fluorescein isothiocyanate	FITC	BioMedica Diagnostics	4508CJ
TF	Mouse	HTF-1	Tissue Factor	Phycoerythrin	PE	BD Pharmingen	550312
Anx5	–	–	Phosphatidylserine	Allophycocyanin	APC	BD Biosciences	550474
IgG1,ҡ	Mouse	MOPC-21	n.a	Phycoerythrin	PE	BD Pharmingen	555749
IgG1,ҡ	Mouse	MOPC-21	n.a	Fluorescein isothiocyanate	FITC	BioLegend	400107

### Culture of mesenchymal stem cells and enrichment of MSC-derived EVs

Human MSCs were isolated from adipose tissue within 8 h after liposuction as previously described^44^. The use of human tissue was approved by the Ethics Committee of the University of Lübeck (Reference number 20-333; date of approval: November 4, 2020), and written informed consent was obtained from the donor. MSCs were cultivated in MEM α supplemented with 0.5% gentamycin, 2.5% human platelet lysate (0.2 μm sterile filtered; Minisart syringe filter), and 1 IU/mL heparin in humidified atmosphere (37 °C, 5% CO_2_). After reaching confluence, cells were detached by accutase treatment, two T175 flasks (Sarstedt, Nümbrecht, Germany) were seeded each with 8.4 × 10^6^ cells in 28 mL MEM a supplemented with 10% human AB serum (centrifuged at 20,000×*g* for 30 min at 4 °C and 0.2 µm sterile filtered; Minisart syringe filter) and cultivated for 6 h. MSCs were pelleted by centrifugation at 300×*g* for 5 min at room temperature for flow cytometric characterization (see below). The remaining supernatant was centrifuged (1500×*g*, 15 min, 4 °C) to remove cell debris, and EVs were pelleted at 20,000×*g* (30 min, 4 °C) using a Sorvall Evolution RC ultracentrifuge, Rotor SS-34 (Thermo Fisher Scientific, Waltham, MA)^20,45^. The pellet was washed with 13 mL DPBS^−/−^ (0.2 µm sterile filtered, Minisart syringe filter), re-centrifuged at 20,000×*g* (30 min, 4 °C), re-suspended in 170 µL DPBS^−/−^ (0.2 µm sterile filtered, Minisart syringe filter), aliquoted, and stored at − 80 °C until flow cytometric analysis of EVs. A detailed workflow on the isolation of MSC-derived EVs is given in Supplementary Fig. S2 online. Relevant EV isolation controls (size distribution and particle concentration) were determined using Nanoparticle Tracking Analysis (NTA; ZetaView, PMX-110, Particle Metrix, Inning, Germany) and are summarized in Supplementary Table S4 online.

### Flow cytometric characterization of TF expression on MSCs

To analyze TF expression on MSCs, 1 × 10^5^ MSCs in 100 µL DPBS^+/+^ were stained with APC-conjugated anti-CD73 (final staining concentration 233 ng/mL for the VD8 panel or 205 ng/mL for the HTF-1), PE-Cy7-conjugated anti-CD105 (final staining concentration 233 ng/mL for the VD8 panel or 205 ng/mL for the HTF-1) and FITC-conjugated anti-TF (clone VD8, isotype G1, final staining concentration 4.7 µg/mL) at different F/P ratios (7.7:1 *vs*. 6.6:1 *vs*. 5.2:1), or with PE-conjugated anti-TF (clone HTF-1; isotype IgE1, final concentration 2 µg/mL) for 15 min in the dark at 22 °C. Antibody panels and staining protocols are given in Supplementary Table S5 online. Cells were pelleted for 1 min at 400×*g*, re-suspended in 500 µL DPBS^+/+^, and characterized by flow cytometry (CytoFLEX LX). Data were acquired for 3 min at a flow rate of 30 μL/min. The respective isotype controls for primary human monocytes and MSCs are shown in Supplementary Fig. S3 online.

### Plasma samples from COVID-19 patients

Plasma samples were obtained from intensive care patients suffering from COVID-19 who required mechanical ventilation (Department of Internal Medicine, Hospital St. Vinzenz, Zams, Austria) between November 2020 and January 2021 (12 patients; time course; 134 samples obtained in total; the results of the main study regarding platelet-monocyte complexes in these samples has been published elsewhere^46^). Sample collection was approved by the Ethics Committee of the Medical University of Innsbruck (Reference number 1144/2020, date of approval: May 20, 2020). The study was conducted in accordance with the declaration of Helsinki and guidelines of good clinical practice as well as local standard operating procedures. Freshly drawn whole blood (S-Monovette® K3 EDTA, Sarstedt, Nümbrecht, Germany) was centrifuged at 2000×*g* (15 min, 22 °C), and plasma was stored at – 80 °C until flow cytometric analysis of EVs (see below).

### Flow cytometric characterization of TF expression on EVs

Prior to staining, purified monocyte- and MSC-derived EVs were diluted 6.7-fold and 500-fold, in 0.1 µm sterile filtered (Millex-VV, Merck Millipore, Tullagreen, Ireland, Catalogue# SLVV033RS) Anx5 binding buffer, respectively. 100 µL of prediluted EV samples were incubated for 30 min in the dark on ice with APC-conjugated Anx5 (BD Biosciences; final staining concentration 37 ng/mL for panels with VD8 or 33 ng/mL for HTF-1) to confirm phosphatidylserine expression and either FITC-conjugated mouse anti-human anti-TF (clone VD8, isotype IgG1, final staining concentration 1.9 µg/mL, different F/P ratios as indicated) or PE-conjugated mouse anti-human anti-TF (clone HTF-1; isotype IgG1, final concentration 1.7 µg/mL). After incubation, samples were further diluted with 400 µL filtered Anx5 binding buffer and kept on ice in the dark until analysis. To remove eventual precipitates, all fluorochrome conjugates were centrifuged at 18,600×*g* for 10 min at 4 °C prior to use. Fluorescent silica beads (1 μm, 0.5 μm, 0.1 μm; excitation/emission 485/510 nm; Kisker Biotech, Steinfurt, Germany) were used to define the analysis window consistent with the size of EVs (Supplementary Fig. S4 online). The triggering signal for EVs was set to the violet side scatter (405 nm), and the gate was set below the 1 µm bead cloud as previously described^47,48^ and shown in Supplementary Fig. S4a online. EVs were identified as Anx5-binding events. Acquisition was performed for 2 min at a flow rate of 10 µL/min. To prevent swarm detection while maintaining significant particle counts, serial sample dilutions were performed (Supplementary Fig. S5 online). The laser specifications of the CytoFLEX LX, the instrument settings for acquisition, as well as the compensation matrices are given in Supplementary Tables S6–S8 online. Data were analyzed using the Kaluza Software 2.1.

Assay controls, including buffer-only controls for Anx5 staining, unstained controls, isotype controls, as well as single stained controls are shown in Supplementary Fig. S4b online. Buffer with reagent controls for anti-TF clone VD8 with different F/P ratios were performed to control for potential differences in EV concentrations (Supplementary Fig. S6 online). The presence of intact EVs was confirmed by detergent lysis with 0.25% Triton-X 100 (Supplementary Fig. S7 online). Triton-X 100 was added to EV-containing samples for 5 min in the dark on ice following antibody staining as described above. Further details on the flow cytometric characterization of EVs are reported according to the MIFlowCyt-EV framework^28^ (Supplementary Table S9 online) and the MIFlowCyt guidelines^49^ (Supplementary Table S10 online).

Plasma samples were diluted 1:100 in Anx5 binding buffer, and EVs in 100 µL diluted sample were stained with APC-conjugated Anx5 (final staining concentration 37 ng/mL for panels with VD8 or 33 ng/mL for HTF-1) as marker for EVs exposing phosphatidylserine, PE-Cy7-conjugated anti-CD41 (final staining concentration 925 ng/mL for panels with VD8 or 847 ng/mL for HTF-1) as platelet marker, PB-conjugated anti-CD45 (final staining concentration 1.9 µg/mL for panels with VD8 or 1.7 µg/mL for HTF-1; all Beckman Coulter) as leukocyte marker, FITC-conjugated anti-CD235a (final staining concentration 9 µg/mL) as red blood cell marker (Invitrogen, Waltham, MA), and FITC-conjugated anti-TF (clone VD8, isotype G1, final staining concentration 1.9 µg/mL, different F/P ratios as indicated) or PE-conjugated anti-TF (clone HTF-1; isotype IgE1, final concentration 1.7 µg/mL). Prior to analysis, stained samples were diluted 1:5 in 0.1 µm sterile filtered Anx5 binding buffer, and flow cytometric characterization was carried out as described above. Details on the used antibody panels and the staining protocols are included in Supplementary Table S5 online. A detailed workflow of the characterization of EV samples is included in Supplementary Fig. S2 online.

### Competition assays

Monocyte- or MSC-derived EVs were pre-stained with FITC-conjugated anti-TF (clone VD8, F/P ratio 5.2:1), followed by staining with anti-TF (clone VD8, F/P ratio 7.7:1) for 30 min on ice. For comparison, staining was performed with FITC-conjugated anti-TF (clone VD8, F/P ratio 7.7:1) alone, and flow cytometric analysis was performed as described above.

### Statistical analysis

Statistical analysis was performed using GraphPad Prism version 7.02 (La Jolla, CA). The Friedman test followed by Dunn´s multiple comparisons test was used to compare multiple groups. Data are presented as mean ± standard deviation (SD) and significance was accepted at *p* ≤ 0.05.

### Supplementary Information

Supplementary Information.

## Data Availability

Data are available on request from the corresponding author. Flow cytometric data are uploaded to the FlowRepository and are accessible via the following link: http://flowrepository.org/id/FR-FCM-Z6N6.
