# Supplementary material for: The fluorochrome-to-protein ratio is crucial for the flow cytometric detection of tissue factor on extracellular vesicles
Source: Sci Rep. 2024 Mar 17;14:6419. doi: 10.1038/s41598-024-56841-5 (PMC10944842; doi:10.1038/s41598-024-56841-5)
Supplement: Supplementary file 1 — Supplementary Information. [file 41598_2024_56841_MOESM1_ESM.pdf]

Supplementary Material

The Fluorochrome-to-Protein Ratio is Crucial for the Flow Cytometric Detection of Tissue Factor on Extracellular Vesicles

René Weiss[1,+], Marwa Mostageer[1,+], Tanja Eichhorn[1], Silke Huber[2], Dominik Egger[3], Andreas Spittler[4], Carla Tripisciano[5], Cornelia Kasper[3], Viktoria Weber[1,*]

[1] Center for Biomedical Technology, Department for Biomedical Research, University for Continuing Education Krems, Krems, Austria
[2] Institute of Hygiene and Medical Microbiology, Medical University of Innsbruck, Innsbruck, Austria
[3] Institute of Cell and Tissue Culture Technology, Department of Biotechnology, University of Natural Resources and Life Sciences, Vienna, Austria
[4] Core Facility Flow Cytometry & Surgical Research Laboratories, Medical University of Vienna, Vienna, Austria
[5] Clinical Division of Haematology and Haemostaseology, Department of Medicine I, Medical University of Vienna, Vienna, Austria

[+] Equally contributing first authors

***** Corresponding author: Prof. Viktoria Weber, University for Continuing Education Krems, Dr.-Karl-Dorrek-Strasse 30, 3500 Krems, Austria, phone: +43 2732 893 2632; viktoria.weber@donau-uni.ac.at

Supplementary Table S1. Concentrations for monocyte-derived EVs.

	VD8						HTF-1	
	F/P ratio 7.7:1		F/P ratio 6.6:1		F/P ratio 5.2:1			
No.	EVs/µL*	TF^+ EVs/µL	EVs/µL	TF^+ EVs/µL	EVs/µL	TF^+ EVs/µL	EVs/µL	TF^+ EVs/µL
1	2.64×10^4	2.13×10^3	2.54×10^4	1.47×10^3	2.36×10^4	7.17×10^1	2.26×10^4	2.06×10^2
2	1.67×10^4	1.45×10^3	1.65×10^4	1.13×10^3	1.65×10^4	1.05×10^2	2.63×10^4	3.26×10^2
3	3.71×10^4	2.21×10^3	4.66×10^4	1.71×10^3	4.83×10^4	1.11×10^3	3.12×10^4	3.07×10^2

* Anx5-binding events.

Supplementary Table S2. Concentrations for MSC-derived EVs.

	VD8						HTF-1	
	F/P ratio 7.7:1		F/P ratio 6.6:1		F/P ratio 5.2:1			
No.	EVs/µL*	TF^+ EVs/µL	EVs/µL	TF^+ EVs/µL	EVs/µL	TF^+ EVs/µL	EVs/µL	TF^+ EVs/µL
1	1.71×10^6	2.07×10^5	1.66×10^6	1.09×10^5	1.46×10^6	8.62×10^4	1.39×10^6	1.87×10^4
2	1.31×10^6	1.05×10^5	1.51×10^6	6.67×10^4	1.27×10^6	4.50×10^4	8.05×10^5	6.25×10^2
3	3.00×10^6	3.40×10^5	3.08×10^6	1.37×10^5	2.70×10^6	8.24×10^4	2.45×10^6	9.23×10^3
4	1.85×10^6	1.63×10^5	1.86×10^6	1.21×10^5	1.80×10^6	6.32×10^4	1.55×10^6	7.43×10^3
5	1.52×10^6	4.04×10^5	1.30×10^6	1.94×10^5	1.33×10^6	6.12×10^4	1.36×10^6	1.35×10^4

* Anx5-binding events.

Supplementary Table S3. EV concentrations for COVID-19 plasma.

	VD8						HTF-1	
	F/P ratio 7.7:1		F/P ratio 6.6:1		F/P ratio 5.2:1			
**No.**	**EVs/μL***	**TF^+ EVs/μL**	**EVs/μL**	**TF^+ EVs/μL**	**EVs/μL**	**TF^+ EVs/μL**	**EVs/μL**	**TF^+ EVs/μL**
**1**	1.86×10^5	3.04×10^4	1.84×10^5	1.04×10^4	1.89×10^5	2.41×10^3	1.27×10^5	3.15×10^2
**2**	3.40×10^4	6.05×10^3	3.38×10^4	1.65×10^3	3.55×10^4	4.55×10^2	2.57×10^4	1.35×10^2
**3**	3.71×10^4	9.05×10^3	3.42×10^4	2.67×10^3	3.19×10^4	1.85×10^2	1.62×10^4	1.00×10^2
**4**	1.97×10^4	4.18×10^3	1.72×10^4	1.10×10^3	1.64×10^4	4.00×10^2	1.05×10^4	8.50×10^1
**5**	6.11×10^4	1.86×10^4	6.03×10^4	1.37×10^4	6.12×10^4	1.45×10^3	4.50×10^4	4.80×10^2
**6**	8.82×10^4	2.16×10^4	7.62×10^4	1.31×10^4	9.80×10^4	4.08×10^3	6.24×10^4	5.65×10^2
**7**	6.44×10^4	1.83×10^4	6.98×10^4	6.26×10^3	6.88×10^4	1.51×10^3	5.95×10^4	4.15×10^2
**8**	4.14×10^4	7.45×10^3	3.95×10^4	6.46×10^3	3.96×10^4	3.63×10^3	2.57×10^4	1.15×10^2
**9**	1.20×10^5	2.38×10^4	1.14×10^5	7.70×10^3	1.14×10^5	1.77×10^3	7.80×10^4	3.85×10^2
**10**	2.30×10^5	4.08×10^4	2.25×10^5	1.21×10^4	1.95×10^5	2.54×10^3	1.34×10^5	4.00×10^2

* Anx5-binding events.

Supplementary Table S4. EV size distribution and particle concentration measured by nanoparticle tracking analysis (NTA).

	Monocyte-derived EVs		MSC-derived EVs	
**No.**	**Size [nm]**	**Concentration [particles/mL]**	**Size [nm]**	**Concentration [particles/mL]**
**1**	121 ± 3	$3.60 \pm 0.65 \times 10^{10}$	158 ± 5	$3.28 \pm 0.66 \times 10^{11}$
**2**	175 ± 4	$2.12 \pm 0.63 \times 10^{10}$	171 ± 2	$3.41 \pm 0.09 \times 10^{11}$
**3**	162 ± 16	$1.14 \pm 0.18 \times 10^{10}$	182 ± 1	$2.62 \pm 0.12 \times 10^{11}$

Supplementary Table S5. Antibody panels and staining protocols used in this study for staining monocytes, mesenchymal stem cells (MSCs), and extracellular vesicles (EVs). Volumes refer to the used stock solution in a final volume of 100 µL sample. For catalogue numbers of the individual fluorochrome-conjugates, please refer to Table 1 in the main manuscript. MM, Mastermix; Anx5, annexin V; VD8, anti-tissue factor clone VD8; HTF-1, anti-tissue factor clone HTF-1.

**Monocytes**					
**MM for VD8**	**Vol.**	**Stock Concentration**	**MM for HTF-1**	**Vol.**	**Stock Concentration**
CD14 - PE	5 µL	1 µg/mL	CD14 - PB	2 µL	200 µg/mL
CD66b - APC	5 µL	12.5 µg/mL	CD66b - APC	5 µL	12.5 µg/mL
CD45 - PB	5 µL	100 µg/mL	CD41 - PE-Cy7	1 µL	50 µg/mL
CD41 - PE-Cy7	1 µL	50 µg/mL	HTF-1 - PE	20 µL	12.5 µg/mL
VD8 - FITC	5 µL	100 µg/mL			
**Monocyte-derived EVs**					
**MM for VD8**	**Vol.**	**Stock Concentration**	**MM for HTF-1**	**Vol.**	**Stock Concentration**
CD41 - PE-Cy7	2 µL	50 µg/mL	Anx5 - APC	2 µL	2 µg/mL
CD45 - PB	2 µL	100 µg/mL	HTF-1 - PE	16 µL	12.5 µg/mL
Anx5 - APC	2 µL	2 µg/mL			
VD8 - FITC	2 µL	100 µg/mL			
**MSCs**					
**MM for VD8**	**Vol.**	**Stock Concentration**	**MM for HTF-1**	**Vol.**	**Stock Concentration**
CD73 - APC	1 µL	25 µg/mL	CD73 - APC	1 µL	25 µg/mL
CD105 - PE-Cy7	1 µL	25 µg/mL	CD105 - PE-Cy7	1 µL	25 µg/mL
VD8 - FITC	5 µL	100 µg/mL	HTF-1 - PE	20 µL	12.5 µg/mL
**MSC-derived EVs**					
**MM for VD8**	**Vol.**	**Stock Concentration**	**MM for HTF-1**	**Vol.**	**Stock Concentration**
Anx5 - APC	2 µL	2 µg/mL	Anx5 - APC	2 µL	2 µg/mL
VD8 - FITC	2 µL	100 µg/mL	HTF-1 - PE	16 µL	12.5 µg/mL
**COVID-19 plasma**					
**MM for VD8**	**Vol.**	**Stock Concentration**	**MM for HTF-1**	**Vol.**	**Stock Concentration**
CD41 - PE-Cy7	2 µL	50 µg/mL	Anx5 - APC	2 µL	2 µg/mL
CD45 - PB	2 µL	100 µg/mL	HTF-1 - PE	16 µL	12.5 µg/mL
Anx5 - APC	2 µL	2 µg/mL			
VD8 - FITC	2 µL	100 µg/mL			
**COVID-19 plasma EV origin**					
**MM**	**Vol.**	**Stock Concentration**			
CD41 - PE-Cy7	2 µL	50 µg/mL			
CD235a - FITC	2 µL	0.5 mg/mL			
CD45 - PB	2 µL	100 µg/mL			
Anx5 - APC	2.5 µL	2 µg/mL			

Supplementary Table S6. Laser specifications of the flow cytometer CytoFLEX LX.

Laser	Wavelength [nm]	Power [mW]	Filter
Violet	405	80	450/45 (PB)
Blue	488	50	525/40 (FITC)
Yellow Green	561	30	585/42 (PE)<br>763/42 (PE-Cy7)
Red	638	50	660/10 (APC)

Supplementary Table S7. Instrument settings of the flow cytometer CytoFLEX LX. The detector gain for the individual channels and the Threshold (Trigger Level) are given. FS, forward scatter; SS, side scatter; H, height.

	**Monocytes**		**Monocyte-derived EVs**		**MSCs**		**MSC-derived EVs**		**COVID-19 plasma**		
	**Tissue Factor**										**EV-origin**
	**VD8**	**HTF-1**	**VD8**	**HTF-1**	**VD8**	**HTF-1**	**VD8**	**HTF-1**	**VD8**	**HTF-1**	
**FS**	39	39	348	448	24	24	348	448	348	448	348
**SS**	31	31	40	40	16	16	40	40	40	40	40
**Violet SS**			16	16			16	16	16	16	16
**525/40 FITC**	761		200		100		200		200		200
**763/43 PE-Cy7**	731	731	600		111	111	600		600		600
**660/10 APC**	362	362	736	736	258	258	736	736	736	736	736
**450/45 PB**	42	42	200				200		200		200
**585/42 PE**	113	250		100		100		100		100	300
**Threshold (Trigger Level)**	FS 10,000 (H)	FS 10,000 (H)	Violet SS 2000 (H)	Violet SS 2000 (H)	FS 10,000 (H)	FS 10,000 (H)	Violet SS 2000 (H)	Violet SS 2000 (H)	Violet SS 2000 (H)	Violet SS 2000 (H)	Violet SS 2000 (H)

Supplementary Table S8. Compensation matrices for the flow cytometric analysis of cells and EVs.

	Cells					EVs				
	**FITC**	**PE**	**PC7**	**APC**	**PB**	**FITC**	**PE**	**PC7**	**APC**	**PB**
**FITC**	-	10.85	0.8	0	0	-	0.8	0.2	3.5	0
**PE**	0	-	0.5	0	0	0.4	-	1.45	0	0
**PC7**	0	1.54	-	10	0	0	1.4	-	4	0
**APC**	0	0	0	-	0	0	0	0	-	0
**PB**	0	0	0.1	0	-	0	0	0	0	-

Supplementary Table S9. MIFlowCyt-EV framework.

Framework Criteria	What to report	Please complete each criterion
1.1 Preanalytical variables conforming to MISEV guidelines.	Preanalytical variables relating to EV sample including source, collection, isolation, storage, and any others relevant and available in the performed study.	All relevant pre-analytical variables are given in the Methods Section.
1.2 Experimental design according to MIFlowCyt guidelines.	EV-FC manuscripts should provide a brief description of the experimental aim, keywords, and variables for the performed FC experiment(s) using MIFlowCyt checklist criteria: 1.1, 1.2, and 1.3, respectively. Template found at www.evflowcytometry.org.	The experimental aim (to assess the influence of the F/P ratio on TF detection on EVs) and the relevant variables are described in the Introduction and Methods Section of our manuscript. The relevant keywords are given.
2.1 Sample staining details	State any steps relating to the staining of samples. Along with the method used for staining, provide relevant reagent descriptions as listed in MIFlowCyt guidelines (Section 2.4 Fluorescence Reagent(s) Descriptions).	A detailed workflow of the characterization of EV samples (from isolation to flow cytometry) is given in Supplementary Figure S2. Fluorochrome conjugates are listed in Table 1. The staining protocol is given in Supplementary Table S5.
2.2 Sample washing details	State any steps relating to the washing of samples.	Please refer to the Methods Section and to the workflow in Supplementary Figure S2.
2.3 Sample dilution details	All methods and steps relating to sample dilution.	Please refer to the Methods Section and to the workflow in Supplementary Figure S2.
3.1 Buffer alone controls.	State whether a buffer-only control was analyzed at the same settings and during the same experiment as the samples of interest. If utilized it is recommended that all samples be recorded for a consistent set period of time e.g. 5 minutes, rather than stopping analysis at a set recorded event count e.g. 100,000 events. This allows comparisons of total particle counts between controls and samples.	Please refer to Supplementary Figure S4 for buffer-only controls. Buffer-only controls were used for setting up the protocol only.
3.2 Buffer with reagent controls.	State whether a buffer with reagent control was analyzed at the same settings, same concentrations, and during the same experiment as the samples of interest. If used state what the results were.	Please refer to Supplementary Figure S4 for buffer with reagent controls. Buffer with reagent controls were used for setting up the protocol only.
3.3 Unstained controls.	State whether unstained control samples were analyzed at the same settings and during the same experiment as stained samples. If used, state what the results were, preferably in standard units.	See point 3.2.
3.4 Isotype controls.	The use of isotype controls is applicable to immunofluorescence labelling only. State whether isotype controls were analyzed at the same settings and during the same experiment as stained samples. If utilized, state which antibody they are matched to, the concentration used, and what the results were (Section 4.2, 4.3, 4.4). Due to conjugation differences between manufacturers if should be stated if the isotype controls are from the same manufacturer as the matched antibodies.	Isotype controls are listed in Table 1 of the main manuscript and data are shown in Supplementary Figure S4. They were used for establishing the protocol only.<br><br>For tissue factor detection on EVs the following antibody clones with the respective isotype controls have been used:

		1) Anti-TF clone VD8-FITC (Biomedica Diagnostics; Ref. 4508CJ; 100 µg/mL) *vs.* IgG1,κ Isotype Control-FITC (BioLegend; Ref. 400107; 0.5 mg/mL)<br>2) Anti-TF clone HTF-1-PE (BD Pharmingen; Ref. 550312; 12.5 µg/mL) *vs.* IgG1,κ Isotype Control-PE (BD Pharmingen; Ref. 555749; 50 µg/mL)
3.5 Single-stained controls.	State whether single-stained controls were included. If used state whether the single-stained controls were recorded using the same settings, dilutions, and during the same experiment as stained samples and state what the results were, preferably in standard units (Section 4.2, 4.3, 4.4).	See point 3.2.
3.6 Procedural controls.	State whether procedural controls were included. If used, state the procedure and if the procedural controls were acquired at the same settings and during the same experiment as stained samples.	Not required.
3.7 Serial dilutions.	State whether serial dilutions were performed on samples and note the dilution range and manner of testing. The fluorescence and/or scatter signal intensity would ideally be reported in standard units (see Section 4.3, 4.4) but arbitrary units can also be used. This data is best reported by plotting the recorded number events/concentration over a set period of time at different sample dilution. The median fluorescence intensity at each of the dilutions should also ideally be plotted on the same or a separate plot.	Serial dilutions were performed to determine the optimal dilution in order to avoid "swarm detection" (Supplementary Figure S5).
3.8. Detergent treated EV-samples	State whether samples were detergent treated to assess lability. If utilized, state what detergent was used, the end concentration of the detergent, and what the results were of the lysis.	Please refer to the Methods Section and to Supplementary Figure S7.
4.1 Trigger Channel(s) and Threshold(s).	The trigger channel(s) and threshold(s) used for event detection. Preferably, the fluorescence calibration (Section 4.3) and/or scatter calibration (Section 4.4) should be used in order to report the trigger channel(s) and threshold(s) in standardized units.	Please refer to Supplementary Table S7.
4.2 Flow Rate / Volumetric quantification.	State if the flow rate was quantified/validated and if so, report the result and how they were obtained.	Please refer to the Methods Section.
4.3 Fluorescence Calibration.	State whether fluorescence calibration was implemented, and if so, report the materials and methods used, catalogue numbers, lot numbers, and supplied reference units for the standards. Fluorescence parameters may be reported in standardized units of MESF, ERF, or ABC beads. The type of regression used, and the resulting scatter plot of arbitrary data vs standard data for the reference particles should be supplied.	Quality control beads (CytoFLEX Daily QC Fluorospheres; Ref. B53230, Beckman Coulter) were run prior to each measurement. Since we performed a direct comparison of the anti-tissue factor clone VD8 with three different fluorochrome-to-protein (F/P) ratios, fluorescence calibration was not required.

4.4 Light Scatter Calibration.	State whether and how light scatter calibration was implemented. Light scatter parameters may be reported in standardized units of nm2, along with information required to reproduce the model.	Methods Section<br><br>Calibration was performed with fluorescent silica beads (0.1 µm, 0.5 µm, 1 µm; excitation/emission 485/510; Kisker Biotech).
5.1 EV diameter/surface area/volume approximation.	State whether and how EV diameter, surface area, and/or volume has been calculated using FC measurements.	Not performed.
5.2 EV refractive index approximation.	State whether the EV refractive index has been approximated and how this was done.	Not performed.
5.3 EV epitope number approximation.	State whether EV epitope number has been approximated, and if so, how it was approximated.	Not performed.
6.1 Completion of MIFlowCyt checklist.	Complete MIFlowCyt checklist criteria 1 to 4 using the MIFlowCyt guidelines. Template found at www.evflowcytometry.org.	See Supplementary Table S10.
6.2 Calibrated channel detection range	If fluorescence or scatter calibration has been carried out, authors should state whether the upper and lower limits of a calibrated detection channel were calculated in standardized units. This can be done by converting the arbitrary unit scale to a calibrated scaled, as discussed in Section 4.3 and 4.4, and providing the highest unit on this scale and the lowest detectable unit above the unstained population. The lowest unit at which a population is deemed 'positive' can be determined a variety of ways, including reporting the 99th percentile measurement unit of the unstained population for fluorescence. The chosen method for determining at what unit an event was deemed positive should be clearly outlined.	Gates were adjusted manually related to the unstained samples or the respective isotype controls.
6.3 EV number/concentration.	State whether EV number/concentration has been reported. If calculated, it is preferable to report EV number/concentration in a standardized manner, stating the number/concentration between a set detection range.	Please refer to Results Section and Supplementary Tables S1-3.
6.4 EV brightness.	When applicable, state the method by which the brightness of EVs is reported in standardized units of scatter and/or fluorescence.	Not performed.
7.1. Sharing of data to a public repository.	Provide a link to the experimental data in a public data repository.	Flow cytometric data are uploaded to the FlowRepository and are accessible via the following link: http://flowrepository.org/id/FR-FCM-Z6N6

Supplementary Table S10. MIFlowCyt checklist.

Requirement	Please Include Requested Information
1.1. Purpose	Investigation of the effect of different fluorochrome-to-protein (F/P) ratios of anti-TF (tissue factor)-fluorochrome conjugates on the flow cytometric detection of TF^+ extracellular vesicles (EVs) from activated monocytes, mesenchymal stem cells (MSCs), and in COVID-19 plasma.
1.2. Keywords	extracellular vesicles, flow cytometry, fluorochrome-to-protein ratio, tissue factor
1.3. Experiment variables	TF expression on EVs was compared among different sources (monocytes *vs.* mesenchymal stem cells *vs.* COVID-19 plasma) using TF-specific antibodies with increasing F/P ratios (see Supplementary Figure S2).
1.4. Organization name and address	University for Continuing Education Krems, Dr.-Karl-Dorrek-Strasse 30, 3500 Krems, Austria.
1.5. Primary contact name and email address	Prof. Viktoria Weber, viktoria.weber@donau-uni.ac.at
1.6. Date or time period of experiment	2022-10-13 – 2023-05-24
1.7. Conclusions	The detection of TF on EVs critically depends on the F/P ratio of the antibody-fluorochrome conjugate, whereas it does not affect the detection of TF on cells.
1.8. Quality control measures	The following quality control measures were performed:<br>Buffer alone controls, Buffer with reagent controls, unstained controls, isotype controls, single stained controls, serial dilutions, and detergent lysis. Calibrated fluorescent silica beads were used to define the analysis window for EVs.
2.1.1.1. (2.1.2.1., 2.1.3.1.) Sample description	For our study, we used monocytes (isolated from human peripheral blood and stimulated with lipopolysaccharide), mesenchymal stem cells (isolated from adipose tissue), and human plasma from COVID-19 patients to characterize cells and their secreted EVs.
2.1.1.2. Biological sample source description	Peripheral blood as the source of monocytes and plasma; adipose tissue as the source of mesenchymal stem cells.
2.1.1.3. Biological sample source organism description	Peripheral blood and adipose tissue from human.
2.1.2.2. Environmental sample location	Not applicable
2.3. Sample treatment description	Refer to the Supplementary Table S5 (Antibody panels and staining protocols) and Supplementary Figure S2 illustrating the workflow on the isolation and characterization of EVs.
2.4. Fluorescence reagent(s) description	Refer to Table 1 of the main manuscript.
3.1. Instrument manufacturer	Beckman Coulter, Brea, CA
3.2. Instrument model	CytoFLEX LX
3.3. Instrument configuration and settings	Refer to Supplementary Table S6 and S7, for laser specifications and instrument settings, respectively.
4.1. List-mode data files	The flow cytometric data files have been uploaded to the FlowRepository and are available as follows:<br>http://flowrepository.org/id/FR-FCM-Z6N6.

4.2. Compensation description	Refer to Supplementary Table S8.
4.3. Data transformation details	For cells linear transformation and for EVs logarithmic transformation was used.
4.4.1. Gate description	The gate was set below the 1 µm bead cloud. EVs were identified as Anx5-binding events in the gate.
4.4.2. Gate statistics	As acquisition software the CytExpert Software 2.4 and for analysis the Kaluza Software 2.1 were used.
4.4.3. Gate boundaries	Calibrated fluorescent silica beads were used to define the analysis window for EVs. Isotype controls were used to set positive expression.

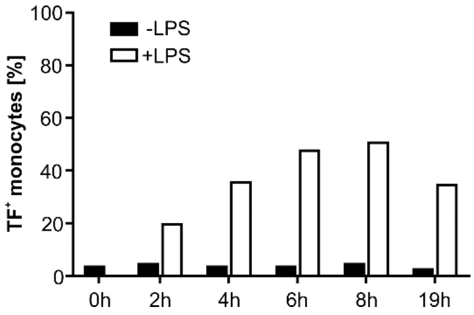

Supplementary Fig. S1. Time-dependent TF expression on isolated monocytes upon LPS stimulation. Isolated monocytes were stimulated with 10 ng/mL LPS (*E. coli*) for 2, 4, 6, 8, and 19 hours (+LPS) or left untreated (-LPS) and TF expression was assessed by staining the cells with FITC-conjugated anti-TF (clone VD8, F/P ratio 5.2:1).

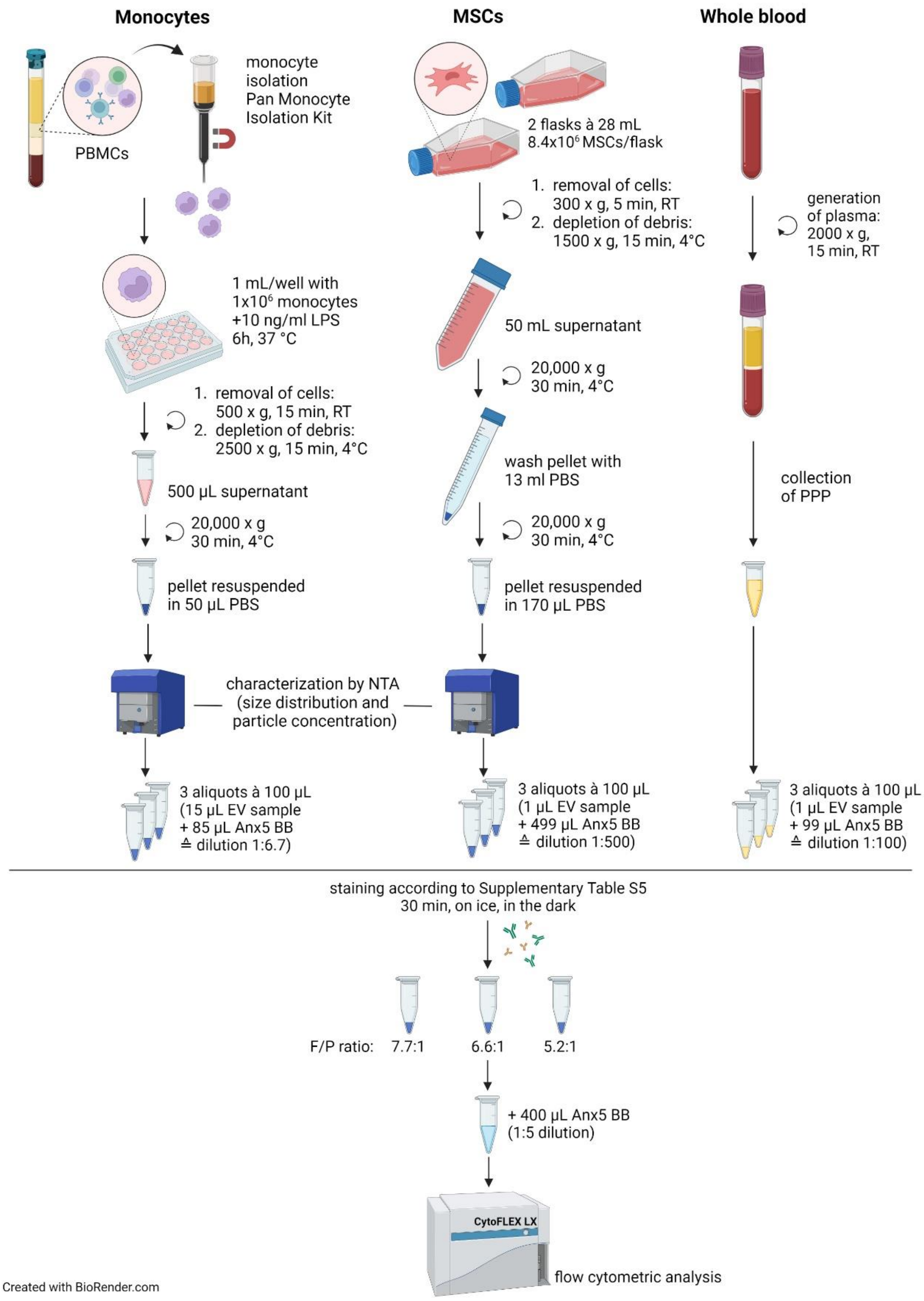

Supplementary Fig. S2. Workflow on the isolation and characterization of extracellular vesicles. PBMCs, peripheral blood mononuclear cells; LPS, lipopolysaccharide; RT, room temperature; PBS, phosphate buffered saline; NTA, nanoparticle tracking analysis; Anx5 BB, Annexin V binding buffer; MSCs, mesenchymal stem cells; PPP, platelet poor plasma; F/P ratio, fluorochrome-to-protein ratio.

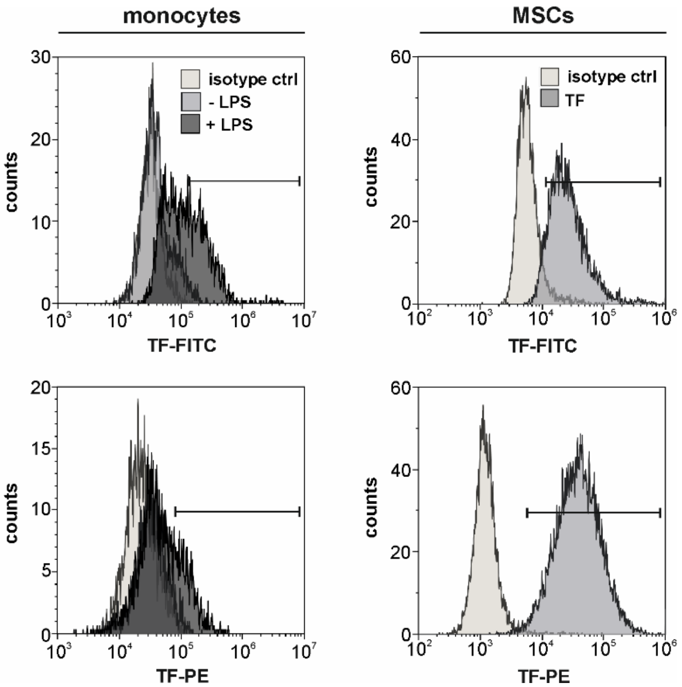

Supplementary Fig. S3. Controls for the flow cytometric characterization of monocytes and MSCs. The respective isotype controls and single stainings with FITC-conjugated anti-TF (clone VD8, upper panel) or PE-conjugated anti-TF (clone HTF-1, lower panel) are shown for unstimulated (-LPS) and stimulated (+LPS) monocytes (left), as well as for MSCs (right).

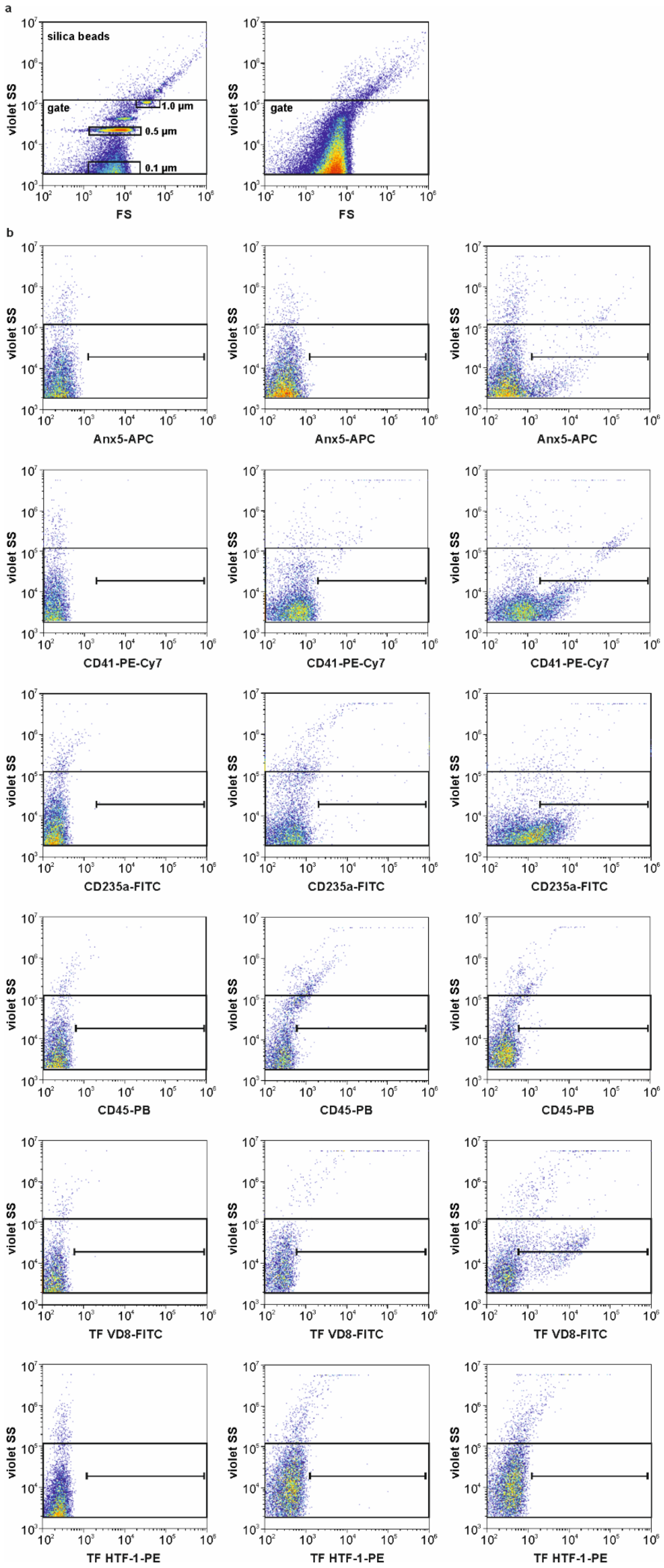
a
silica beads
gate
1.0 µm
0.5 µm
0.1 µm
violet SS
FS
b
Anx5-APC
CD41-PE-Cy7
CD235a-FITC
CD45-PB
TF VD8-FITC
TF HTF-1-PE

Supplementary Fig. S4. Calibration and controls for the flow cytometric detection of extracellular vesicles. (a) Flow cytometric characterization was performed on a CytoFLEX LX flow cytometer (Beckman Coulter), using calibrated fluorescent silica beads (0.1, 0.5, and 1.0 µm) to define the analysis window consistent with the size of EVs. The gate was set below the 1 µm bead cloud as described in the Methods section. Staining of EVs was performed as described in the Methods section and representative forward scatter (FS) *vs*. violet side scatter (violet SS) density plots are shown. **(b)** Unstained controls (left panel), isotype controls (middle panel), and single stainings (right panel) are shown for EV characterization. Anx5 staining in PBS without Ca^{++} and Mg^{++} was used as buffer-only control. Bars indicate positive expression.

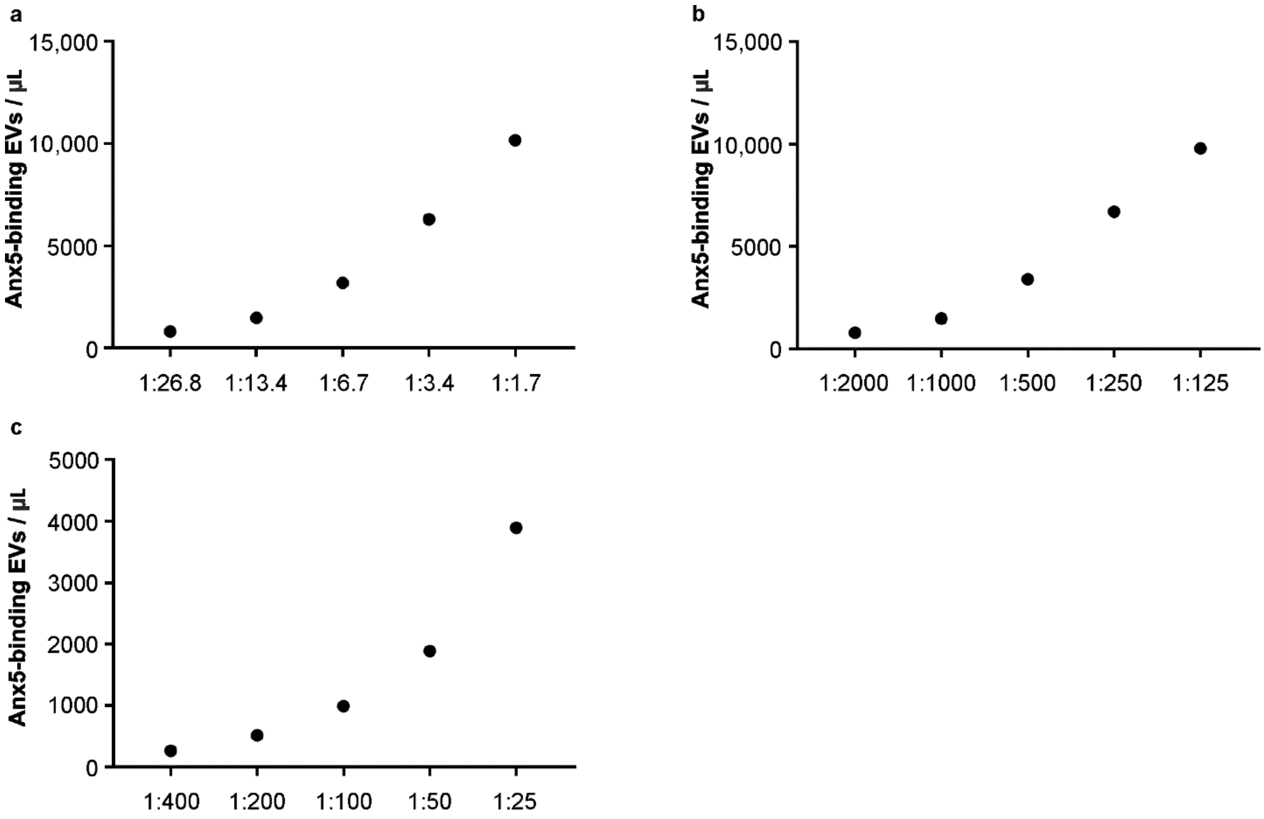

Supplementary Fig. S5. Serial dilutions. Dilution series of **(a)** monocyte-derived EVs, **(b)** MSC-derived EVs, and **(c)** COVID-19 plasma were performed to determine the optimal dilution in order to avoid swarm detection. The optimal dilution factor was 1:6.7 for monocyte-derived EVs, 1:500 for MSC-derived EVs, and 1:100 for COVID-19 plasma.

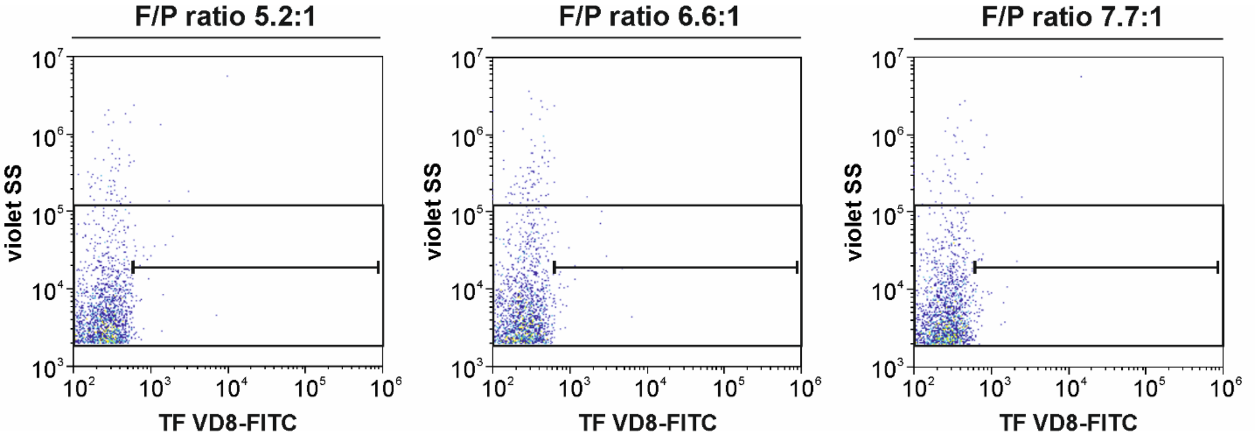

Supplementary Fig. S6. Buffer with reagent controls. Sterile filtered (0.1 µm) Anx5 binding buffer was incubated with FITC-conjugated anti-TF clone VD8 at different F/P ratios to confirm the absence of aggregates in the different antibody batches.

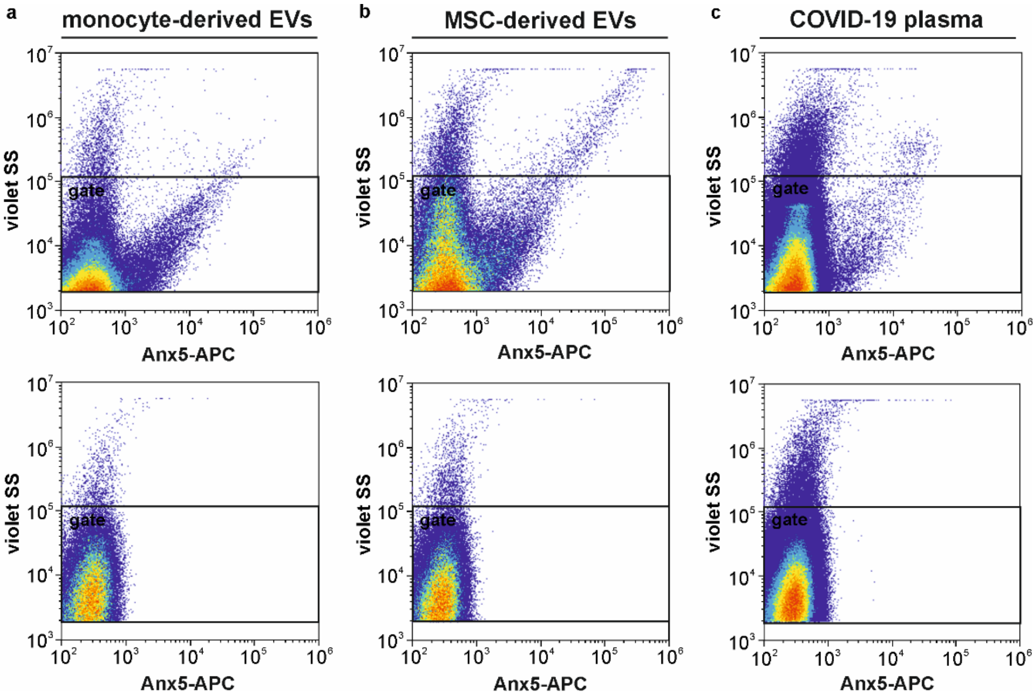

Supplementary Fig. S7. Detergent treatment controls. Detergent lysis with Triton-X 100 abolished the cloud for **(a)** monocyte-derived EVs, **(b)** MSC-derived EVs, and **(c)** EVs in COVID-19 plasma confirming the presence of intact vesicles.
